# ASPECT Score and Its Application to Vasospasm in Aneurysmal Subarachnoid Haemorrhage: a Case–Control Study

**DOI:** 10.1007/s12975-022-01073-w

**Published:** 2022-08-09

**Authors:** Melissa Bautista, Rebecca Burger, Ian A. Anderson, Ryan K. Mathew

**Affiliations:** 1 Department of Neurosurgery, Leeds Centre for Neurosciences, Leeds Teaching Hospitals NHS Trust, G Floor, Leeds General Infirmary, Jubilee Wing Great George Street, Leeds, LS1 3EX UK; 2 School of Medicine, Clinical Sciences Building, Leeds Institute of Medical Research at St James’s, University of Leeds, Room 7.6, Leeds, LS9 7TF UK

**Keywords:** Subarachnoid haemorrhage, Delayed cerebral ischaemia, ASPECT score

## Abstract

Delayed cerebral ischaemia (DCI) is a significant complication of aneurysmal subarachnoid haemorrhage (aSAH) and is strongly associated with poorer outcome. The Alberta Stroke Program Early Computer Tomography (ASPECT) score is an established scoring tool, used in acute ischaemic stroke, to quantify early ischaemic changes on CT head scans. We aim to identify if ASPECT scoring correlates with functional outcome in DCI following aSAH. Retrospective case–control study. Inclusion criteria: admission to the Department of Neurosurgery at Leeds Teaching Hospitals NHS Trust (a tertiary neurosurgical centre in the United Kingdom) between 2014 and 2018, with a diagnosis of anterior circulation aneurysmal subarachnoid haemorrhage; as confirmed by initial CT scan and subsequent CT angiography or catheter digital subtraction angiography. Cases were those who developed DCI (*n* = 43) and controls were randomly selected from those who did not develop DCI (*n* = 46) but otherwise met the same inclusion criteria. The primary outcome measure was Glasgow Outcome Score (GOS): assessed at discharge and 3 months. ASPECT scores were calculated from non-contrast CT head scans by three researchers blinded to each other and clinical outcome. Spearman’s rank correlation was used to calculate correlation between ASPECT scores and GOS. ASPECT score positively correlated with GOS in the cases both at discharge (Spearman rho 0.436, *p* = 0.003) and at 3 months (Spearman rho 0.431, *p* = 0.004). When corrected for Fisher grading, the adjusted odds ratio of having a high GOS with a low ASPECT score at discharge was OR 0.74 (95% CI 0.61–0.94, *p* = 0.003), and 3 months OR 0.73 (95% CI 0.59–0.91, *p* = 0.005). ASPECT score significantly correlates with clinical outcome in DCI post aSAH, even after correcting for Fisher grade. ASPECT scoring may identify patients at risk of poor outcome following DCI and represents a quick and reliable tool that aids in clinical decision-making and prognostication.

## Introduction

Aneurysmal subarachnoid haemorrhage (aSAH) is the spontaneous rupture of an intracranial aneurysm, leading to deposition of blood within the subarachnoid space. aSAH is responsible for 5% of all strokes and 85% of atraumatic subarachnoid haemorrhages [1] aSAH is a significant cause of mortality and morbidity; there is a pre-hospital mortality rate of approximately 15% [2, 3] with an overall [4] case fatality of around 50%. Of those who survive, over 50% will have long-term psychological and neurological impairments [5, 6].

Of the many potential complications of a SAH, one of the most significant is vasospasm [7]. This term applies to the radiological diagnosis of mechanical cerebral arterial narrowing as a result of vessel reaction to subarachnoid blood. Irritation by blood products in the subarachnoid space causes the activation of potent vasoconstrictors causing cerebral arteries to narrow, thus reducing the blood delivery to cerebral tissue and potentially leading to irreversible ischaemic damage [8]. Vasospasm can affect up to 70% of patients and commonly presents between days 5–14 post-ictus. The clinical manifestation of vasospasm is referred to as delayed cerebral ischaemia (DCI). Signs of DCI include new focal neurological deficits and/or a reduction in conscious level not attributed to any other cause [9] DCI is a significant complication of SAH and is associated with a poor outcome [10].

The Alberta Stroke Programme Early Computer Tomography (ASPECT) score is a 10-point scoring tool used to quantify early ischaemic changes on CT scans in acute anterior ischaemic strokes [11]. This score provides a systematic approach to quantify the degree of middle cerebral artery (MCA) territory ischaemic changes on non-contrast CT head images. ASPECT score has shown to correlate with prognosis, where a score of less than 7 is associated with severe disability or death at 3 months [12].

ASPECT score also identifies those at risk of symptomatic intracranial haemorrhage following thrombolysis [12]. It provides a standardised radiological assessment of ischaemic changes and provides an objective quantification of the severity of ischaemia on CT [13]. ASPECT score has previously been validated in the literature and is shown to be superior to the previous 1/3 MCA rule [13] and its use has been extended to the triage of patients for the appropriate management [14].

Since DCI represents an acute ischaemic process, we hypothesise that ASPECT score will also be applicable to this population, and that a high ASPECT score will correlate with better functional outcome.

## Methods

**Fig. 1:**
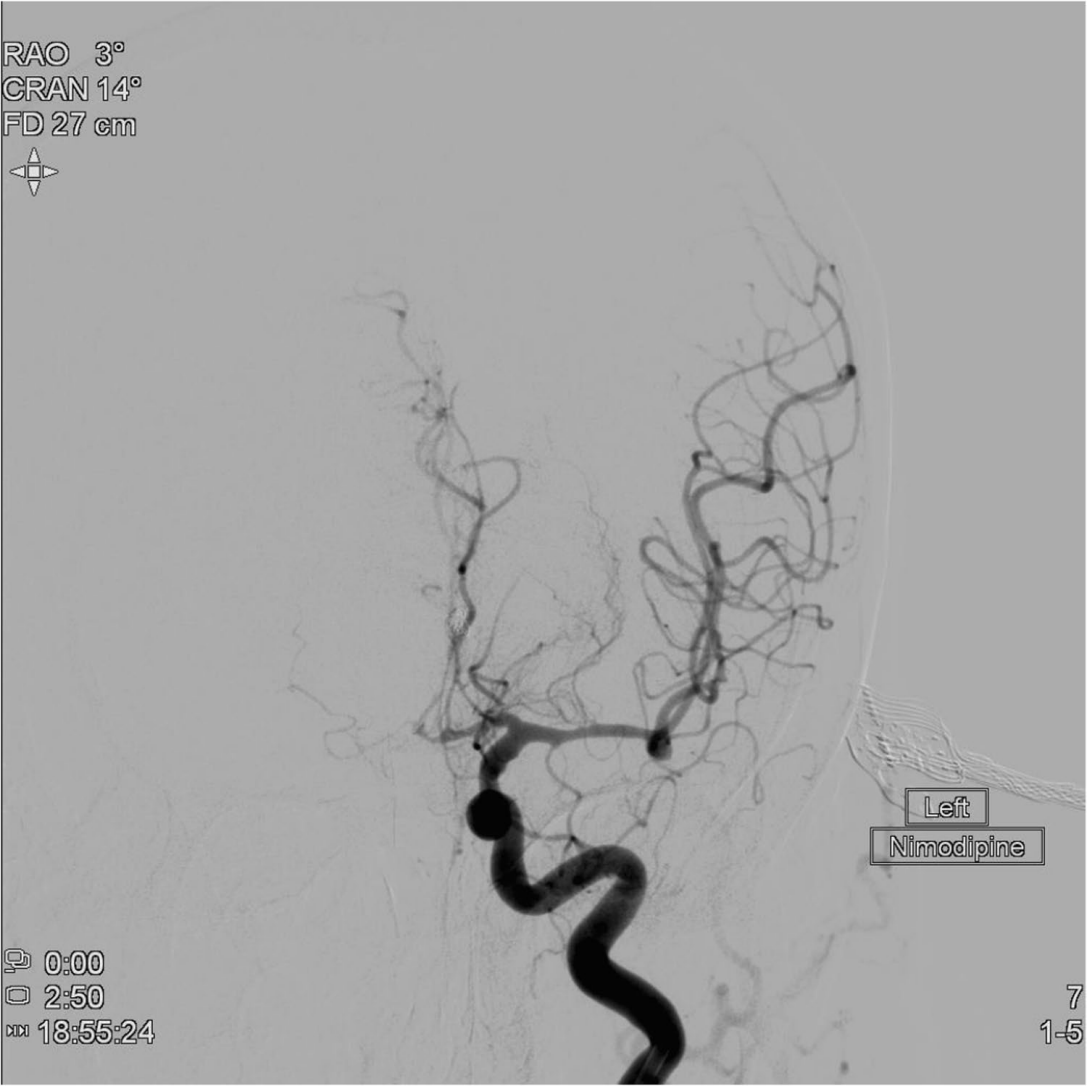
AP radiograph from digital subtraction angiography (left internal carotid artery injection), performed for patient with subarachnoid haemorrhage and radiographic vasospasm

The authors completed a retrospective case–control study. The inclusion criteria were patients admitted to a single neurosurgical centre in UK with aSAH (due to an anterior circulation aneurysm) between 2014 and 2018, confirmed by CT scan and CT angiography or catheter digital subtraction angiography. Exclusion criteria included all non-aneurysmal cases of SAH, those below the age of 18 and those with non-anterior circulation aneurysms. Cases were defined as those who developed clinical signs of DCI (i.e. new neurological deficit or reduction in consciousness) with vasospasm confirmed on angiographic imaging during their admission (*n* = 43), and controls were randomly selected from those who were admitted during the same time period with aSAH (due to an anterior circulation aneurysm), but did not develop clinical signs of DCI and had no evidence of vasospasm on imaging during their admission (*n* = 46). Controls were selected using a random number generator. An example of a vasospasm case from this series is depicted in Fig. 1.

**Fig. 2:**
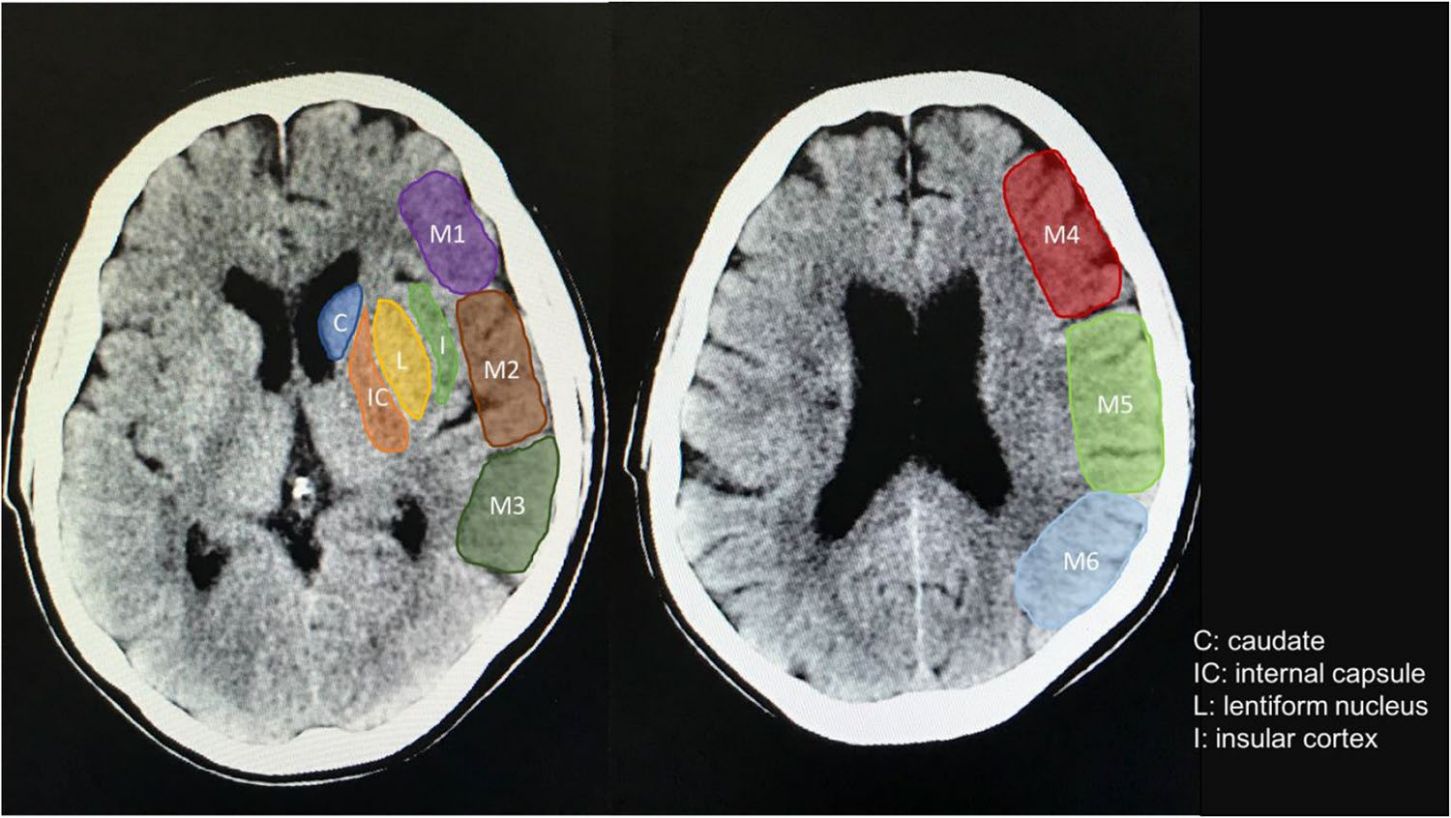
Calculating ASPECT score from non-contrast CT head images. (**a**) Level of the basal ganglia for visualisation of caudate, internal capsule, insula, and M1–3. (**b**) Level of the corona radiata for visualisation of M4–6

For the case group, the ASPECT score was calculated from the non-contrast CT head (5 mm slice thickness) obtained at the onset of DCI and for the controls, the score was calculated from a CT head (5 mm slice thickness) undertaken at least 24 h following admission. Where multiple CT scans were available for the same patient, the scan performed closest to day 6 post-ictus (i.e. in the peak vasospasm timeframe) was selected. All scans used in the study were analysed using Patient Archiving and Communication Systems imaging software. Scoring was conducted by taking away a single point (starting from 10), for signs of ischaemia (loss of grey-white differentiation, hypoattenuation) within the 10 areas of the middle cerebral artery (MCA) territory (max score 10 = no ischaemia, min score 0 = ischaemia in all 10 MCA areas). The scores were calculated from two levels within the CT image. The first level was at the basal ganglia which allowed for visualisation of 7 areas of the MCA territory (caudate, internal capsule, insula, and M1–3). The second level was at the corona radiata which includes MCA territories M4–6 (Fig. 2).

ASPECT scores were calculated by three observers (MB, RB, IAA) blinded to each other and the outcome. The initial inter-observer agreement between MB and RB was kappa 0.43, with an inter-observer agreement of 83% with a 1-point tolerance applied. Those ASPECT scores where disagreement was noted were calculated by a third observer (IAA) (a neurosurgical consultant with subspecialty neurovascular interest) and final agreement was obtained through discussion. There were no cases in which a final consensus agreement could not be reached.

### Statistical Analysis

Statistical analysis was conducted using SPSS for Windows, version 10. *p* < 0.05 was considered statistically significant. The Mann–Whitney test was used to compare between groups, due to the ordinal nature of GOS. Potential confounding variables were identified by univariate analysis, with the effect of these further investigated using an ordinal logistic regression model for the multivariate analysis. A backward selection procedure was used to retain only statistically significant variables in the final model.

## Results

A total of 89 (43 cases, 46 controls) patients were included in this study. The demographics are demonstrated in Table 1. There was no significant difference in demographics between cases and controls. The median age of SAH onset in our population was 53 years. 75% of patients were female.

**Table 1:** Patient demographics between case and control groups

	Patients	Controls	*p* value
Age (mean)	53 (32–70)	56 (32–78)	0.085
Sex			0.57
Male	11	11	
Female	32	35	
Smoking			0.34
Yes	12	20	
No	18	20	
Unknown	13	6	
Hypertension			0.58
Yes	10	15	
No	20	25	
Unknown	13	6	
Ischaemic heart disease			0.38
Yes	1	0	
No	30	38	
Unknown	12	8	
Treatment of aneurysm			0.8
Endovascular	37	43	
Microsurgical	5	0	
Combined	1	0	
None	0	3	
WFNS grade			0.19
1	17	22	
2	11	11	
3	2	3	
4	5	6	
5	8	4	
Fisher grade			0.38
1	0	1	
2	0	1	
3	14	23	
4	29	21	

Days 6 and 7 post-ictus demonstrated the highest frequencies of DCI in our case group with 42% (18/43) of patients developing DCI on these days. The median World Federation of Neurosurgical Societies (WFNS) score for cases and controls was 2 (*p* = 0.19). Comorbidities such as hypertension and ischaemic heart disease were equivalent between those who developed DCI and those who did not (*p* = 0.58 and *p* = 0.38). All of the cases had a Fisher score of 3, while for the controls 2% scored 1 (1/46), 2% scored 2 (1/46), 87% scored 3 (40/46) and 9% scored 4 (4/46). There was no difference in Fisher score between cases and controls (*p* = 0.38).

The majority of patients in both groups received endovascular aneurysm management (Table 2).

**Table 2:** Aneurysmal management type in cases and controls

Management	Cases (*n* = 43)	Controls (*n* = 46)	Difference (*p* value)
Endovascular	37	42	*p* = 0.80
Microsurgical	5	0	
Combination	1	0	
None	0	3	

Controls had higher GOS scores, with 81% scoring 5 at discharge, compared to 33% of cases (*p* < 0.001). There was a mortality rate of 23% (10/43) in DCI group, compared to 6% (3/46) in the control group. Both at discharge and at 3 months, the median GOS was 3 for cases and 5 for controls (*p* ≤ 0.001) (Table 3).

**Table 3:** GOS in cases and controls

Outcome	GOS score	Controls *n* (%)	Cases *n* (%)	*p* value
GOS at discharge	1	3 (6%)	10 (23%)	** < 0.001**
	2	0 (0%)	2 (5%)	
	3	5 (11%)	10 (23%)	
	4	1 (2%)	7 (16%)	
	5	38 (81%)	14 (33%)	
GOS at 3 months	1	4 (9%)	11 (25%)	** < 0.001**
	2	0 (0%)	2 (5%)	
	3	2 (4%	8 (19%)	
	4	3 (7%)	7 (16%)	
	5	36 (80%)	15 (35%)	

Bold typface is used to highlight differences achieving statistical significance

### ASPECT Score in Cases and Controls

Median ASPECT score for cases was 6 and 10 for controls (*p* ≤ 0.001). ASPECT score positively correlated with GOS in cases at discharge, with odds ratio 1.34 (95% CI 1.09–1.65, *p* = 0.005), and at 3 months (odds radio 1.33, 95% CI 1.08–1.62, *p* = 0.006).

Univariate analysis demonstrated both WFNS grade and ASPECT score to have a significant relationship with GOS in cases at discharge (Table 4).

**Table 4:** Univariate analysis of relationship between variables and GOS for cases at discharge

Variable	Odds ratio (95% CI)	*p* value
Sex	0.91 (0.27, 3.01)	0.88
Hypertension	1.94 (0.45, 8.37)	0.38
Smoking status	0.44 (0.11, 1.71)	0.24
Age	0.74 (0.39, 1.38)	0.34
Aneurysm treatment 1*	2.10^ (0.42, 10.5)	0.37
Aneurysm treatment 2**	2.01^ (0.47, 8.56)	0.35
WFNS grade	0.47 (0.31, 0.70)	** < 0.001**
Fisher grade	-	0.24
ASPECT score	1.34 (1.09, 1.65)	**0.005**

Bold typface is used to highlight differences achieving statistical significance

(-) Unable calculate odds ratio as all cases have the same Fisher score

(*) Microsurgical treatment of aneurysm

(**) Combined endovascular and microsurgical treatment

(^) OR relative to just endovascular treatment

A multivariate analysis was performed to examine the joint association of variables with GOS. A backwards selection procedure was used to retain only statistically significant variables for the final model. This demonstrated that ASPECT score, WFNS grade and increasing age (in 10 year increments) were all independently associated with GOS score at discharge. A higher ASPECT score was associated with a higher GOS score, which each 1-point increase in ASPECT score increasing the odds of being in the next highest GOS score by 30% (Table 5).

**Table 5:** Multivariate analysis of relationship between variables and GOS for cases at discharge

Variable	Odds ratio (95% CI)	*p* value
Age	0.42 (0.20, 0.87)	**0.02**
WFNS grade	0.45 (0.29, 0.71)	**0.001**
ASPECT score	1.30 (1.03, 1.64)	**0.03**

Bold typface is used to highlight differences achieving statistical significance

Similar analysis was performed at 3 months post discharge. Univariate analysis similarly demonstrated ASPECT score had a relationship approaching significance with and WFNS score a significant relationship with GOS (*p* = 0.06 and *p* = 0.004 respectively), with again a higher ASPECT score associated with higher GOS, and a higher WFNS grade associated with lower GOS.

## Discussion

The age distribution and comorbidities of patients presenting with aSAH in our cohort reflect that seen in the existing literature [15–17]. This suggests that the cases within our study are representative of the broader population of patients with aSAH. Within our study population, those who developed DCI post aSAH had worse functional outcome than those who suffered aSAH but did not develop DCI, again a finding that is supported elsewhere in the literature [18, 19].

The previous validation of ASPECT scoring is for acute ischaemic strokes in MCA territory infarcts; there is strong evidence supporting the correlation between ASPECT score, ischaemic volume and patient outcomes within this context. The findings of this study suggest that ASPECT score may have wider application and that radiological evidence of ischaemic changes is related to functional outcome in patients with DCI post aSAH.

We have demonstrated that ASPECT score correlates significantly with functional outcome in patients with DCI, at both discharge and after 3 months. This remains true, even when controlling for other known variables linked to outcome in this context. By calculating ASPECT score from non-contrast CT head images obtained at time of neurological deterioration, it may therefore be possible to better prognosticate on eventual functional outcome. This could potentially aid clinical decision-making and the planning of rehabilitation and psychosocial support for patients and their families.

aSAH (and therefore DCI) is a disease largely affecting individuals of a working age and, given the significant mortality and morbidity associated with aSAH, the disease causes a significant socioeconomic burden [20]. Substantial future work is required to better understand, treat or even eventually prevent DCI, since this represents a source of potentially treatable or preventable morbidity and mortality in this patient group. It is important that cases can be objectively quantified for severity.

This paper represents the first of its kind, the first to have objectively quantified the radiological extent of DCI and to have correlated this significantly with clinical outcome. This may improve our understanding of DCI in future but also allow for standardised, objective comparisons to be made between future studies and patient cohorts.

## Limitations

The calculation of ASPECT score has often demonstrated an inter-observer variability, and we demonstrated a degree of variability between the two initial observers (kappa 0.43). This suggests ASPECT score calculation may be subject to variable interpretation. In order to minimise the effects of variability, the authors introduced a third observer in cases of non-agreement and the remaining differences were resolved through discussion.

ASPECT score in clinical practice, as calculated by a fully qualified radiologist, has been demonstrated to have good inter-observer variability and is more likely to reflect the external validity of the scoring tool [21].

As a retrospective study, this work is subject to the limitations associated with retrospective work. While control cases were not demonstrated to have been significantly different to the cases, they were identified through a random number generator and only those with an appropriate CT scan were included. Not all patients with aSAH will go on to have further CT scans during their admission; these would tend only to be performed due to a relevant clinical indication such as to monitor for hydrocephalus or to assess for the cause of a clinical change. It follows that these controls may therefore represent a subset of the available cohort that were, in some way or other, less well than those that never had further CT scans performed during their admission. While this is a methodological and statistical limitation, logic would dictate that, if anything, the findings of this study would be perhaps even stronger if a more general selection of the available non-cases had been possible.

The small sample size within the study, which is a product of the relatively rare outcome being studied, may also represent a potential limitation. However, despite these methodological limitations, this study suggests scope for further research and the application of ASPECT score in the context of DCI.

## Summary

ASPECT score correlates with functional outcome in patients with DCI post aneurysmal subarachnoid haemorrhage. It assesses early ischaemic changes on non-contrast CT head images and suggests radiological quantification of ischaemia in DCI is related to functional outcome. It represents a quick and reliable assessment tool which can aid in clinical assessment and prognostication in DCI following aSAH.

## Abbreviations

DCI: Delayed cerebral ischaemia
aSAH: Aneurysmal subarachnoid haemorrhage
ASPECT: Alberta Stroke Program Early Computer Tomography
CT: Computer tomography
MRI: Magnetic resonance imaging
GOS: Glasgow outcome score
DSA: Digital subtraction angiography
WFNS: World Federation of Neurosurgical Societies
MCA: Middle cerebral artery
